# Transcriptome Profile of the Asian Giant Hornet (*Vespa mandarinia*) Using Illumina HiSeq 4000 Sequencing:* De Novo* Assembly, Functional Annotation, and Discovery of SSR Markers

**DOI:** 10.1155/2016/4169587

**Published:** 2016-01-10

**Authors:** Bharat Bhusan Patnaik, So Young Park, Se Won Kang, Hee-Ju Hwang, Tae Hun Wang, Eun Bi Park, Jong Min Chung, Dae Kwon Song, Changmu Kim, Soonok Kim, Jae Bong Lee, Heon Cheon Jeong, Hong Seog Park, Yeon Soo Han, Yong Seok Lee

**Affiliations:** ^1^Department of Life Science and Biotechnology, College of Natural Sciences, Soonchunhyang University, 22 Soonchunhyangro, Shinchang-myeon, Asan, Chungchungnam-do 31538, Republic of Korea; ^2^Trident School of Biotech Sciences, Trident Academy of Creative Technology (TACT), Chandrasekharpur, Bhubaneswar, Odisha 751024, India; ^3^National Institute of Biological Resources, 42 Hwangyeong-ro, Seo-gu, Incheon 22689, Republic of Korea; ^4^Korea Zoonosis Research Institute (KOZRI), Chonbuk National Institute, 820-120 Hana-ro, Iksan, Jeollabuk-do 54528, Republic of Korea; ^5^Hampyeong County Insect Institute, Hampyeong County Agricultural Technology Center, 90 Hakgyohwasan-gil, Hakgyo-myeon, Jeollanam-do 57158, Republic of Korea; ^6^Research Institute, GnC BIO Co., Ltd., 621-6 Banseok-dong, Yuseong-gu, Daejeon 34069, Republic of Korea; ^7^College of Agriculture and Life Sciences, Chonnam National University, 77 Yongbong-ro, Buk-gu, Gwangju 61186, Republic of Korea

## Abstract

*Vespa mandarinia* found in the forests of East Asia, including Korea, occupies the highest rank in the arthropod food web within its geographical range. It serves as a source of nutrition in the form of Vespa amino acid mixture and is listed as a threatened species, although no conservation measures have been implemented. Here, we performed* de novo* assembly of the* V. mandarinia* transcriptome by Illumina HiSeq 4000 sequencing. Over 60 million raw reads and 59,184,811 clean reads were obtained. After assembly, a total of 66,837 unigenes were clustered, 40,887, 44,455, and 22,390 of which showed homologous matches against the PANM, Unigene, and KOG databases, respectively. A total of 15,675 unigenes were assigned to Gene Ontology terms, and 5,132 unigenes were mapped to 115 KEGG pathways. The zinc finger domain (C2H2-like), serine/threonine/dual specificity protein kinase domain, and RNA recognition motif domain were among the top InterProScan domains predicted for* V. mandarinia* sequences. Among the unigenes, we identified 534,922 cDNA simple sequence repeats as potential markers. This is the first transcriptomic analysis of the wasp* V. mandarinia* using Illumina HiSeq 4000. The obtained datasets should promote the search for new genes to understand the physiological attributes of this wasp.

## 1. Introduction

*Vespa mandarinia*, the Asian giant hornet, is among the largest eusocial wasps globally and is found in Korea, Japan, Taiwan, and China, as well as the Indian subcontinent. These wasps prefer dense woodlands and mountains, where they build their colonies and prey on honeybees, larger insects, other wasps, and hornets [1, 2]. They can cause serious damage to apiaries, with a group of 10–20* V. mandarinia* workers being reported to be able to destroy a population of 10,000 to 30,000 bees in a colony [3]. As an apex predator, they have no natural enemies (except for the endoparasitoid* Xenos moutoni*), and humans pose the biggest threat to them due to their economic value as a source of food (served as hornet sashimi). Habitat destruction due to deforestation has placed this species under threat of extinction, which is exacerbated by the lack of proactive conservation initiatives.

The significant economic value of this species is associated with a substance called Vespa amino acid mixture (VAAM), which has been shown to increase swimming endurance and to decrease lactate and increase glucose concentrations after swimming in mice. This substance is sold as a nutritional supplement to improve athletic performance [4]. One study demonstrated that, with regular exercise, VAAM supplements could increase aerobic fitness while decreasing intra-abdominal fat in humans [5]. Additionally,* V. mandarinia* venom is potent enough to cause the skin to hemorrhage and potentially to cause organ failure [6]. The venom is rich in bioactive peptides and high-molecular weight proteins, such as enzymes, allergens, and toxins [7, 8].

Despite the economic prospects of this species and the need to conserve it in its natural habitat, very little information about the sequence and function of its genes has been revealed. Currently, the NCBI resource for* V. mandarinia* contains only the mitochondrial protein sequences, silk proteins 1 to 5, prepromastoparan, immune responsive protein 30, 14-3-3 zeta, ecdysone receptor, period beta, vespakinin, and mastoparan peptide sequences. Both vespakinin (12 amino acids) and mastoparan (14 amino acids) peptides, isolated from* V. mandarinia* venom, can cause cell degranulation [9, 10]. This limited genetic information is insufficient for conducting a high-throughput functional analysis to unravel the physiological processes associated with the feeding, reproduction, and other behaviors of this wasp. Information on the whole genome sequence of this species would reveal rare gene transcripts, new regulatory elements, alternative splicing, and microsatellites, which would facilitate research on the function of targeted genes.

Whole DNA and RNA sequencing strategies have been highly successful in addressing the genomic challenges in eusocial insects. In particular, transcriptome-wide studies have provided insights into the caste system, and the phenotypic plasticity of the genome has been studied in the Western honey bee,* Apis mellifera* [11]; bumble bee,* Bombus terrestris* [12]; stingless bee,* Melipona quadrifasciata* [13]; and tropical paper wasp,* Polistes canadensis* [14], using conventional and high-throughput sequencing technologies. Furthermore, study in the field of comparative sociogenomics has characterized candidate gene transcripts potentially responsible for behavioral patterns of eusocial insects across lineages [15]. Microarray and digital gene expression signature techniques have also documented differentially expressed genes involved in processes favoring caste-based structures (brain, legs, and ovaries), promoting our understanding of immune-regulatory, stress-signaling, and reproduction pathways [16, 17]. Next-generation sequencing (NGS) technologies and the rapid development of high-throughput platforms have raised the bar regarding the sequencing of nonmodel organisms. These technologies offer a collated and comprehensive output for discovering novel transcripts for functional studies and molecular marker development [18]. Illumina NGS platform-based transcriptomic analysis is reliable for the detection and quantification of active gene transcripts, including novel ones. Illumina HiSeq 2000/2500 platforms have been used for the whole-transcriptome analysis of* Polistes canadensis* [14] and* Cotesia chilonis *[19], venom gland transcriptomic analysis of the Brazilian ant species* Tetramorium bicarinatum* [20], and developmental transcriptomic analysis of* Megalopta genalis* [21], among others.

In this study, we isolated RNA from queens of the Asian giant hornet,* V. mandarinia*, constructed a cDNA library, and conducted whole-transcriptome sequencing using the latest Illumina platform, HiSeq 4000. The sequencer-derived raw reads were preprocessed to quality reads and subsequently processed to contig assembly and unigene clusters using the Trinity* de novo* assembler. For the first time, we report identification of large-scale genomic sequence information and the discovery of microsatellite markers from* V. mandarinia.*

## 2. Materials and Methods

### 2.1. Biological Samples and RNA Extraction

Specimens of* Vespa mandarinia* were collected (on 16 June 2015) at Geomdan Mountain, Sinjang-dong, Hanam-si, Gyeonggi-do Province, South Korea. The sampling for experiments was conducted in accordance with the International Guiding Principles for Biomedical Research Involving Animals (1985) (http://www.ncbi.nlm.nih.gov/books/NBK25438/). Total RNA was isolated from the queens (*n* = 10; whole-body samples) using TRIzol reagent (Invitrogen) following the manufacturer's instructions. The quality of the processed RNA samples was checked using a NanoDrop 2000 spectrophotometer (Thermo, USA) and by running samples on an agarose gel.

### 2.2. Construction of cDNA Library and Illumina HiSeq 4000 Sequencing

RNA samples were sent to GnC Bio Company (Yuseong-gu, Daejeon, South Korea) for mRNA-seq library construction and sequencing using Illumina HiSeq 4000 (Illumina, Inc., San Diego, CA, USA) next-generation platform technology. For mRNA-seq library construction from total RNA, an mRNA-seq sample preparation kit (Illumina Inc., San Diego, CA, USA) was used in accordance with the manufacturer's instructions. Briefly, the total RNA was treated with DNase I and poly-T oligo attached magnetic beads to elute poly-(A^+^) mRNA. The purified mRNA was fragmented using a DNA fragmentation kit (Ambion, Austin, TX, USA) prior to cDNA synthesis. The cleaved mRNA fragments were primed using random-hexamer primers and reverse transcriptase (Invitrogen, Carlsbad, CA, USA) to synthesize first-strand cDNA. The synthesis of second-strand cDNA was accomplished using RNase H (Invitrogen) and DNA polymerase I (New England BioLabs, Ipswich, MA, USA). Subsequently, end-repair of double-stranded cDNA was performed using T4 DNA polymerase, the Klenow fragment, and T4 polynucleotide kinase (New England BioLabs). The end-repaired cDNA was ligated to Illumina paired-end (PE) adapter Oligo-mix with T4 DNA ligase (New England BioLabs) at room temperature for 15 min. Suitable fragments (200 ± 25 bp) were then sequenced in a PE (paired-end) pattern on an Illumina sequencing machine. The sequencing data received from the sequencer were transformed by base calling into raw reads and stored in fastq format.

### 2.3. *De Novo* Assembly of Illumina Sequencing Results

Before performing transcriptome assembly, the raw reads were filtered to remove adapter-only sequences (number of nucleotides of recognized adapter ≤13, and number of nucleotides excluding the adapter ≤35), reads containing unknown nucleotides at a rate of over 5%, and low-quality reads (Phred quality score <20), which could affect the efficiency of assembly. For this purpose, we used the command-line tool Cutadapt (http://code.google.com/p/cutadapt/) with default parameters. The preprocessed clean reads were then used for* de novo* assembly using the short read assembling program Trinity [22], with a *k*-mer size of 25. The Trinity assembler combined reads (with default options and minimum allowed length of 200 bp) to form longer overlapping fragments without gaps, forming contigs. Using the TIGR gene indices clustering tool (TGICL) [23], contigs were assembled into unigenes (having 94% identity, 30 bp overlap). To examine the integrity of the assembly, the unigenes were used as queries in BLASTX against the mitochondrial genome of* V. mandarinia* (GenBank accession KR059904.1).

### 2.4. Annotation of Unigenes

All of the unigene transcripts were used as queries in searches (BLASTX; *E*-value <10^−5^) against protein databases such as PANM (http://malacol.or.kr/blast/) [24], Unigene, Eukaryotic Clusters of Orthologous Groups (KOG), Gene Ontology (GO), and Kyoto Encyclopedia of Genes and Genomes (KEGG) [25] to determine the number of significant hits to known protein sequences in these databases. The numbers of unigene hits to PANM, Unigene, and KOG were visualized using a three-way Venn diagram plot constructed using Venny 2.0 (http://bioinfogp.cnb.csic.es/tools/venny/). BLAST2GO software [26] was used to ascribe GO terms to the unigenes assigned to the categories of biological processes, molecular functions, and cellular components. To identify the most prominent protein domains in the unigene sequences, InterProScan searches were performed remotely from BLAST2GO on the InterProEBI server. The BLAST2GO program can be used to find the top hits for unigene sequences using the BLASTX algorithm, assigning InterPro domains, retrieving all available GO terms for each sequence, and mapping for KEGG pathways. A complete schematic representation of transcriptome assembly workflow for* Vespa mandarinia* is shown in Supplementary Figure  1 in Supplementary Material available online at http://dx.doi.org/10.1155/2016/4169587.

### 2.5. Simple Sequence Repeat Marker Discovery

The microsatellite identification tool (MISA; http://pgrc.ipk-gatersleben.de/misa/) was used to localize the microsatellites in the unigene sequences of* V. mandarinia*. All types of simple sequence repeat (SSR) were identified, from mononucleotide to decanucleotide repeats, including compound SSRs (with more than one type of repeat unit). The selection criteria for the SSRs were as follows: the minimum repeat unit was 10 for mononucleotides 3 for dinucleotides to decanucleotides. Mononucleotide repeats were not considered for the SSR analysis because of the possibility of problems with the Illumina homopolymer sequencing associated with this technology.

## 3. Results and Discussion

### 3.1. Illumina HiSeq 4000 Sequencing and* V. mandarinia* Transcriptome

Illumina NGS platforms have been shown to be suitable for a variety of sequencing applications, including RNA sequencing (RNA-seq), metagenomics, and nucleotide-based marker detection. In RNA-seq applications, Illumina is the preferred choice for* de novo* mapping of transcripts in nonmodel organisms [27, 28]. With HiSeq 4000 instrument, Illumina takes advantage of patterned flow technology for enhanced output of sequence reads/lane, with 1.5Tb PE150 reads in 3.5 days [29]. Using the HiSeq 4000 sequencing platform, we obtained a global overview of the* V. mandarinia* (queen) transcriptome, with over 60 million raw sequences of 9,169,196,254 bases (Table 1). After stringent filtration, 59,184,811 quality reads (Q20 percentage of 99%) were obtained with an accumulated length of 8,297,028,222 bases, representing 97.47% of the raw reads. A summary of the raw sequence processing based on quality parameters is shown in Supplementary Table 1. The adapter trimming analysis suggested that 1.82% of raw sequence bases were discarded, generating 98.18% reads with an average length of 148.3 bp. The sequence data of* V. mandarinia* were deposited in the NCBI sequence read archive (SRA) under accession number SRP064879. All data (transcripts, annotations, and reads) related to the* V. mandarinia* transcriptome are publicly hosted on the local submission webpage (http://bioinfo.sch.ac.kr/submission/). The release date of the information (16 October 2016) is one year after the date of submission (16 October 2015).

The quality reads were assembled into contigs using the Trinity short read assembly program. A total of 147,400 contigs with a mean length of 1,230.9 and an N50 length (contig length such that equally long or longer contigs constitute half of the total assembly length) of 2,578 bp were derived from the* de novo* assembly process. The largest contig assembled was 30,772 bp, and 75,428 contigs were over 500 bp in length. Among the total of 147,400 contigs assembled, 72,077 contigs (48.9%) had lengths varying between 200 and 500 bp, 27,690 (18.79%) between 501 and 1,000 bp, 11,606 (7.87%) between 1,001 and 1,500 bp, and 8,873 (6.2%) between 1,501 and 2,000 bp and 27,154 (18.42%) contigs more than 2,001 bp in length (Supplementary Figure 2(A)). Furthermore, a total of 66,837 unigenes were obtained from the clustering of contigs having a mean length and an N50 length of 1,431.2 and 3,112 bp, respectively. The length of the unigenes varied from 224 to 52,946 bp. Among the 66,837 unigenes clustered from the assembly, 30,668 (45.88%) unigenes were 224 to 500 bp in length, 12,122 (18.14%) were 501–1,000 bp, 5,004 (7.49%) were 1,001 to 1,500 bp, 3,954 (5.92%) were 1,501 to 2,000 bp, and 15,089 (22.58%) unigenes were >2,000 bp in length (Supplementary Figure 2(B)). The mean length of unigenes obtained in this study was found to be significantly greater than that for the transcriptome assemblies of nonmodel species reported elsewhere [16, 30, 31]. We mapped the assembled unigenes against the 13-protein coding genes of* V. mandarinia* mitochondria using BLASTN at a cutoff *E*-value of 1*E* − 5 to clarify the completeness of the Trinity assembly. We found that the mitochondrial-protein coding genes were covered (~100%) by only three assembled unigenes or 0.23 unigene transcripts per gene (Table 2), illustrating the integrity of the assembly. The gene NADH dehydrogenase subunit 5 (ND5) alone was mapped within* V. mandarinia* unigene 01523 (Vm_Uni_01523), whereas seven and five mitochondrial genes were mapped within Vm_Uni_08557 and Vm_Uni_10664, respectively.

### 3.2. Sequence Annotation of Assembled Unigenes

The 66,837 unigene sequences from the assembly of the* V. mandarinia* transcriptome were used as queries for searches against the PANM, Unigene, KOG, GO, and KEGG databases by BLASTX with a cutoff *E*-value of 10^−5^. As shown in Table 3, overall 51,143 unigenes were matched to homologous sequences in the databases, with almost 44,922 sequences being more than 300 bp in length. Of the 66,837 sequences, 44,455 unigenes (66.51%) matched the Unigene database, followed by 40,887 unigenes (61.17%) matching PANM, and 22,390 unigenes (33.5%) matching KOG. The BLASTX results also showed matches of 15,675 (23.45%) and 5,111 unigenes (7.65%) to the GO and KEGG databases, respectively. In addition, our results showed that about 79.65%, 82.63%, and 83.41% of the unigenes that were over 1000 bp in length had BLAST matches in the KOG, GO, and KEGG databases, respectively. It is common for transcripts from transcriptome assemblies not to show homology with known proteins, as they may contain incompletely spliced introns, orphaned untranslated regions, noncoding genes, and random transcriptional noise. PANM has been found to be promising for the annotation of NGS data related to the arthropods, nematodes, and molluscs, as it stands as a filtered format of the NCBI nonredundant protein database (NCBI nr) that provides the more stringent and faster reproduction of annotation results [24].

**Figure 1:**
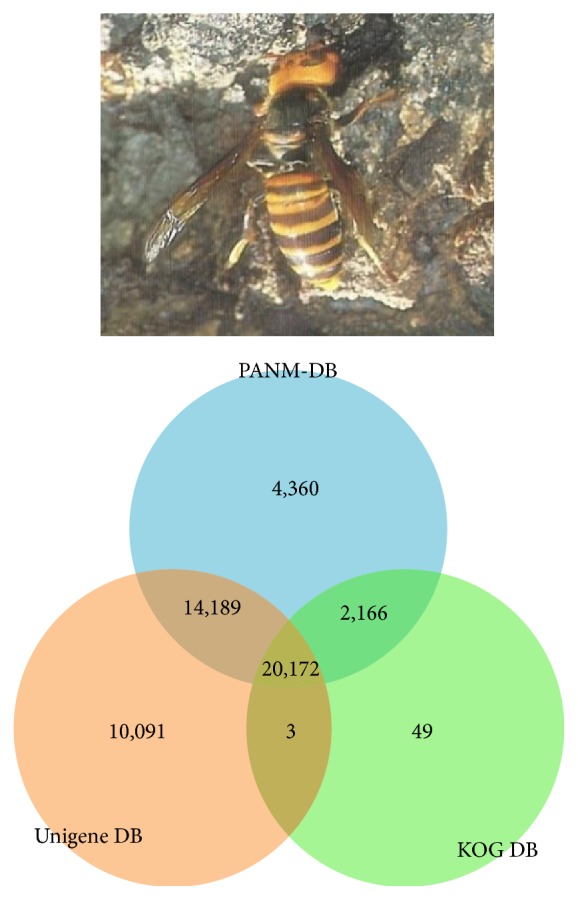
Sequence annotation of unigenes obtained from* V. mandarinia* transcriptome assembly and clustering. A three-way Venn diagram plot shows the unique and overlapped transcripts from unigene annotation hits (BLASTX; *E*-value ≤ 1*E* − 5) against PANM-DB, Unigene, and KOG databases, respectively.

Using a three-way Venn diagram, we studied the overlap of the annotated unigene sequences within each of the PANM, Unigene, and KOG databases (Figure 1). Interestingly, a total of 20,172 unigenes had homologous sequences in all three databases, whereas there were overlaps of 14,189, 2,166, and 3 unigenes between the PANM and Unigene, PANM and KOG, and Unigene and KOG databases, respectively. Presumably, the unigenes that did not show any matching sequence in the databases were short transcripts lacking recognizable protein domains, or they had undergone sequence diversification.

**Figure 2:**
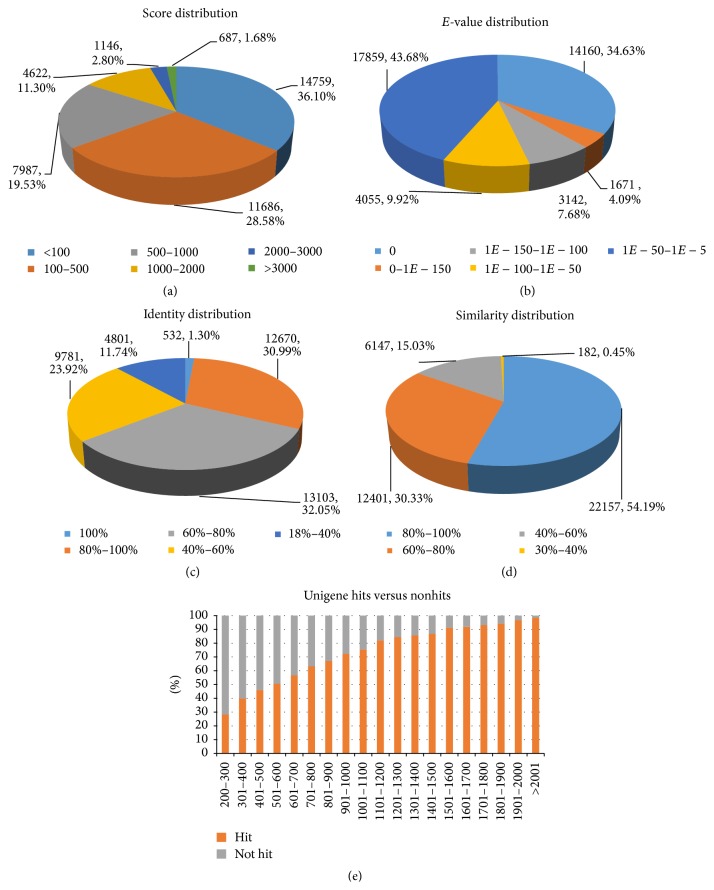
Summary of homology search of assembled unigenes of* V. mandarinia* against PANM-DB. (a) Score distribution of BLASTX hits for each unigene with a cutoff *E*-value of 1*E* − 5. (b) *E*-value distribution of BLASTX hits for each unigene with a cutoff *E*-value of 1*E* − 5. (c) Identity distribution of BLASTX hits for each unigene. (d) Similarity distribution of BLASTX hits for each unigene. (e) Distribution of hit and nonhit sequences as compared with the length of unigenes.

To shed more light on the sequence hits to the PANM database, we studied the results of a homology search conducted using BLASTX (Figure 2). The PANM database was selected as it includes the protein sequences from only the protostome groups and has data processing speed 15 times faster than that of the NCBI nr database [24]. The score distribution showed that 687 unigenes (1.68%) had scores of more than 3000, while the majority of unigenes (14,759, 36.10%) had scores of less than 100 (Figure 2(a)). The *E*-value distribution showed that 17,859 unigenes (43.68%) showed significant homology to the deposited sequences, with *E*-values ranging from 1*E* − 50 to 1*E* − 5 (Figure 2(b)). The identity plot showed a more uniform distribution of the unigenes with 13,103 (32.05%), 12,670 (30.99%), and 9,781 unigenes (23.92%) showing identities of 60–80%, 80–100%, and 40–60%, respectively (Figure 2(c)). The similarity distribution curve showed that the majority of unigene sequences (22,157, 54.19%) exhibited similarities of more than 80%, followed by 30.33% of sequences in the range of 60–80% (Figure 2(d)). In addition, our results showed that unigene sequences with lengths of more than 1,500 bp exhibited significant hits (≥90%) with protein sequences in the PANM database (Figure 2(e)). It can be assumed that the protein-coding genes (especially the more highly expressed ones) give rise to longer and more full-length transcripts.

**Figure 3:**
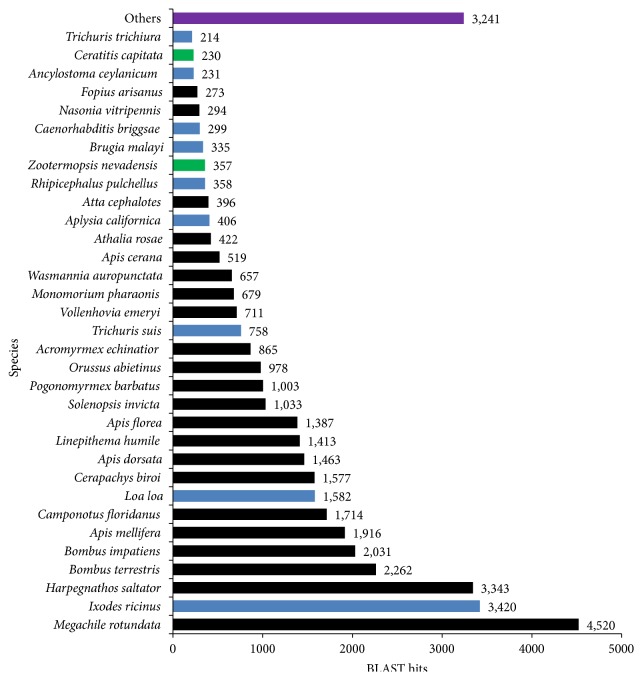
Top-hit species distribution for* V. mandarinia* unigenes against PANM-DB. The top-hit species belonging to class Insecta and order Hymenoptera are shaded black. The top-hit insect species not belonging to order Hymenoptera are shown by green bars. The top-hit species not belonging to class Insecta are shown by blue bars.

The BLASTX top-hit species distribution of the 40,887 unigenes that matched the PANM database showed sequence matches (29,456 sequences, 72.04%) to groups of organisms belonging to the order Hymenoptera, including* Megachile rotundata *(4,520 sequence matches),* Harpegnathos saltator* (3,343),* Bombus terrestris* (2,262),* Bombus impatiens* (2,031), and* Camponotus floridanus* (1,714), and few other members (Figure 3). Some distinct unigene sequences showed hits to noninsect species (7,603 sequences, 18.60%) and other species (3,241 sequences, 7.93%).

### 3.3. Functional Prediction and Classification of Assembled Unigenes

**Figure 4:**
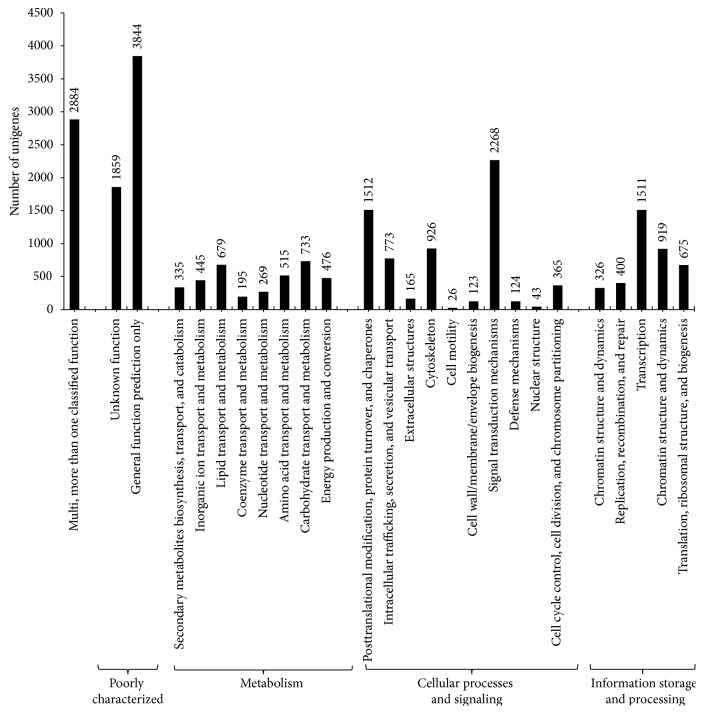
Clusters of Orthologous Groups (KOG DB) based functional analysis of* V. mandarinia* unigenes. The unigenes on the basis of KOG DB analysis are classified to metabolism, cellular processes and signaling, information storage and processing, and poorly characterized gene categories.

To further evaluate the putative functions of the assembled unigenes, we used the unigenes as queries for searches against the KOG database using the BLASTX program. The unigenes were classified into three major categories: “information storage and processing,” “cellular processes and signaling,” and “metabolism,” apart from the “poorly characterized” genes (Figure 4). Out of the 22,390 unigenes annotated to the KOG database, 6,325 (28.25%), 3,831 (17.11%), and 3,647 (16.29%) unigenes were classified into the “cellular processes and signaling,” “information storage and processing,” and “metabolism” categories, respectively. A total of 5,703 (25.47%) unigenes were poorly characterized, and 2,884 (12.88%) unigenes were classified as having more than one functional attribute. Overall, 25 KOG subcategories were represented by the annotated* V. mandarinia* unigenes. The “general function prediction only” (3,844, 17.17%) subcategory formed the largest group, followed by the “multi- (more than one) classified function” subcategory. Among the subcategories belonging to the three major categories, “signal transduction mechanisms” (2,268, 10.13%), “posttranslational modification, protein turnover and chaperones” (1,512, 6.75%), and “transcription” (1,511, 6.75%) formed the larger groups, whereas nuclear structure (43, 0.19%) and cell motility (26, 0.12%) formed the smallest.

**Figure 5:**
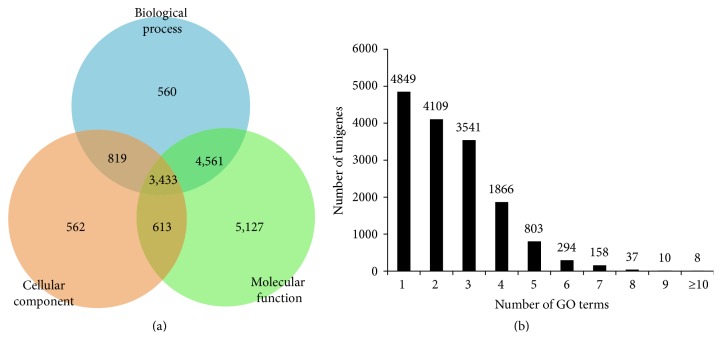
The GO term classification for* V. mandarinia* unigenes. (a) The three-way Venn diagram analysis of unigenes classified into biological process, cellular component, and molecular function categories. (b) The number of unigenes assigned to the number of GO term annotations.

The total unigene profile of* V. mandarinia* was also subjected to BLAST2GO analysis to define the GO term classification and KEGG pathway mapping. The GO annotated sequences were classified into three broad functional categories, namely, biological processes, cellular components, and molecular functions. The distribution of annotated unigenes in the GO functional categories and the number of unigenes ascribed to the GO terms are shown in Figures 5(a) and 5(b), respectively. A total of 13,734 unigenes were ascribed to the molecular function category, along with 9,373 and 5,427 unigenes to the biological process and cellular component categories, respectively. Among all of the unigenes ascribed to each GO functional category, 5,127, 562, and 560 unigenes were unique to the molecular function, cellular component, and biological process categories, respectively. A total of 3,433 unigenes were associated with all three functional categories. Additionally, 7,994 unigenes were associated with both biological processes and molecular functions, 4,252 with biological processes and cellular components, and 4,046 with molecular functions and cellular functions. Moreover, from the total of 15,675 unigenes, 4,849 sequences (30.93%) were assigned to a single GO term, whereas the rest were assigned to more than one functional term. GO functional categories only suggest the grouping of a unigene in terms of its known (or predicted) function, so they are not strictly evidence of functionality. In addition, considering the evidence code distribution associated with each GO annotation (mostly inferred from electronic annotations that were computationally derived), our interpretation of the unigenes relates only to their predicted function.

**Figure 6:**
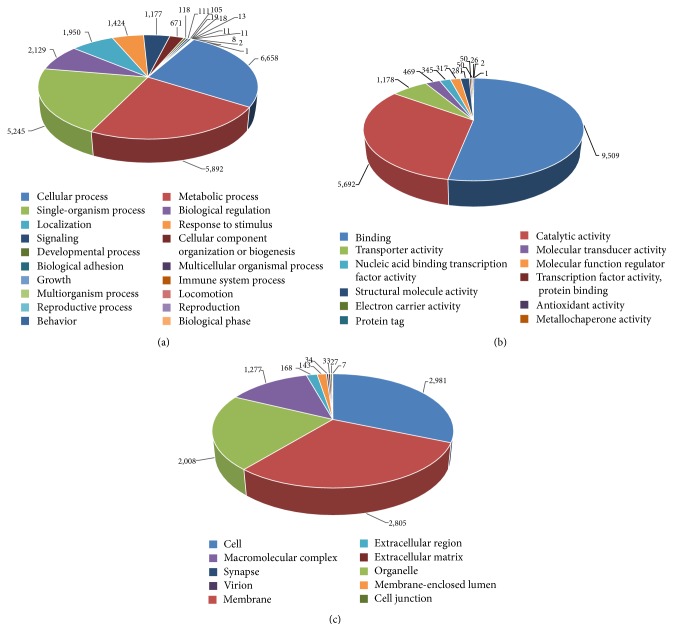
Gene Ontology classifications of assembled unigenes from* V. mandarinia*. (a) Biological processes, (b) molecular function, and (c) cellular components. The number of unigenes ascribed to each subcategory for each classification has been indicated.

Within the biological process category (Figure 6(a)), the majority of transcripts were assigned to cellular processes (6,658 unigenes, 26.05%), followed by metabolic processes (5,892, 23.05%) and single organismal processes (5,245, 20.52%). We found few transcripts that were assigned to GO term categories, such as responses to stimulus (1,424 unigenes, 5.57%), signaling (1,177, 4.60%), immune system processes (19, 0.07%), reproduction (11, 0.04%), and reproductive processes (8, 0.03%). In the molecular function category (Figure 6(b)), the majority of transcripts were assigned to binding (9,509 unigenes, 53.06%) and catalytic activity processes (5,692, 31.76%). A few transcripts were also assigned to other GO terms under categories such as antioxidant activity (26 unigenes, 0.15%) and metallochaperone activity (1, 0.01%). For the transcripts representing the cellular component category (Figure 6(c)), the majority belonged to cell (2,981 unigenes, 31.44%), membrane (2,805, 29.58%), and organelle components (2,008, 21.17%). In a previous study involving a transcriptomic analysis of the Asian honey bee,* Apis cerana cerana*, no unigenes were ascribed to metallochaperone activity, structural molecule activity, and nucleic acid-binding transcription factor-binding activity [32]. Our study can thus be suggested to demonstrate the improved assembly of unigenes annotated to GO functional categories.

**Figure 7:**
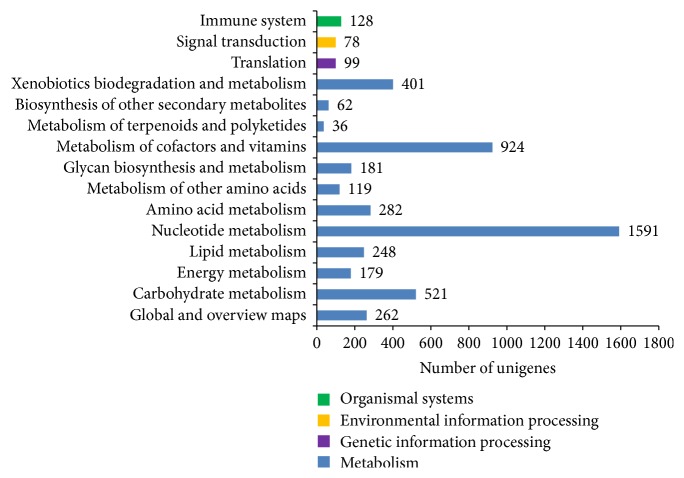
Kyoto Encyclopedia of Genes and Genomes (KEGG) pathway assignment for assembled unigenes from* V. mandarinia*. The unigenes have been categorized to metabolism, genetic information processing, environmental information processing, and organismal systems pathways.

The potential involvement of the assembled* V. mandarinia* unigenes in biological pathways was annotated based on the KEGG database. After a search using 66,837 assembled unigenes queries in KEGG, a total of 5,111 unigenes were assigned to 115 reference canonical pathways in KEGG (Figure 7). The pathways with most unique sequences were metabolic pathways, with 4,806 unigenes (94.03%) associated with basic metabolic functions. The predominant metabolic pathways represented by the unigenes were the amino acid metabolism (1,591), metabolism of cofactors and vitamins (924), and carbohydrate metabolism pathways (521). Other pathways included the immune system (128), translation (99), and signal transduction pathways (78), representing the genetic information processing, environmental information processing, and organismal system pathways of KEGG. The putative KEGG pathways identified provide insights into the specific responses and functions involved in the biological processes of* V. mandarinia*. In the InterProScan analysis, we identified the most prominent protein domains predicted for* V. mandarinia* unigene sequences. A summary of the top InterProScan domains is provided in Table 4. The top three domains predicted are the C2H2-like zinc finger domains, serine/threonine/dual specificity protein kinase domains, and RNA recognition motifs. Other common domains identified are as the WD40 repeat domains, ankyrin repeat domains, and the cytochrome P450 family domains. C2H2-like zinc finger domains are the most prominent DNA-binding motifs present in eukaryotic transcription factors, serving as multiple contacts in binding to DNA [33], RNA, and protein targets [34]. More specifically, they are required as parts of signal transduction, cell growth, differentiation, and development processes. Many of the binding transcription factors are proteins with a tandem array of zinc finger motifs, whereas others have a single zinc finger or multiple split pairs of C2H2 zinc-fingers [35]. The RNA recognition motif domain (about 90 amino acids) is known to bind to single-stranded RNAs including heterogeneous nuclear ribonucleoproteins (hnRNPs), proteins implicated in the regulation of alternative splicing, and proteins that regulate RNA stability and translation. Such motifs were prominently identified in transcriptomic analyses of other insects [36]. The predicted WD40 repeat sequences are involved in signal transduction, cell cycle control, and apoptotic mechanisms [37]. Cytochrome P450 in insects are critical in the metabolism of xenobiotics and confer resistance to synthetic insecticides and plant allelochemicals [38, 39].

### 3.4. Discovery of SSR Markers

In an attempt to characterize the genome and develop new molecular markers, we discovered potential SSR markers from all 66,837 unigenes (95,657,681 bases) assembled from the* V. mandarinia* transcriptome. SSRs are short DNA sequences from 1 to 8 bp in length that are built up by tandem repeats. With the advent and successful execution of NGS technologies, the routine discovery and characterization of SSRs for nonmodel organisms are on the rise [28, 31, 40, 41]. SSR marker discovery and validation are strategic steps for progress in genetic diversity analysis, population genetics, conservation genomics, and association analysis [42]. The lack of genomic resources for* V. mandarinia* and the fact that the species is threatened due to the loss of its habitat indicate the importance of the discovery of SSRs via Illumina HiSeq 4000 sequencing efforts. Among the examined sequences, 62,522 sequences contained SSRs, with 56,378 sequences containing more than one of them (Supplementary Table 2). We identified a total of 534,922 SSRs (defined as mononucleotide to decanucleotide SSRs), with 275,493 SSRs present in compound formation. After the elimination of mononucleotide and decanucleotide repeats, a total of 505,104 SSRs were screened. The frequency and distribution of 505,104 SSRs were analyzed in this study. The distribution of SSRs based on the number of repeat units is shown in Table 5. The dinucleotide repeat motifs were the most abundant (370,450, 73.34%), followed by the trinucleotide (105,010, 20.79%) and tetranucleotide repeats (20,182, 4%). Penta-, hexa-, hepta-, and octanucleotide repeats were less abundant in the assembled unigene sequences. Upon further analysis of the SSR repeat numbers, we found that three tandem iterations were the most common (372,888), followed by four (59,551) and five random iterations (19,793). SSRs with >21 random iterations were more common than SSRs with 12 to 20 random iterations.

**Figure 8:**
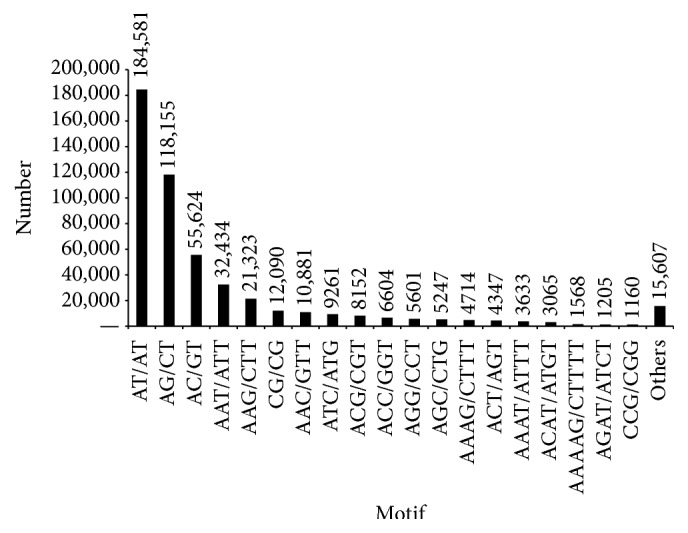
The number of simple sequence repeats (SSRs) discovered in the unigenes of* V. mandarinia* classified based on motif sequence types.

The distribution of the SSRs based on motif sequence types is shown in Figure 8. Overall, 567 motif sequence types were identified, including 2 and 41 repeat types for mononucleotide and decanucleotide repeats, respectively. From among the other repeat types, di-, tri-, tetra-, penta-, hexa-, hepta-, and octanucleotides had 4, 10, 33, 89, 197, 86, and 105 types, respectively. Notably, among the SSR types, AT/AT (184,581, 36.54%) was the most abundant motif, followed by AG/CT (118,155, 23.39%) and AC/GT (55,624, 11.01%) among the dinucleotide repeats, and AAT/ATT (32,434, 6.42%) and AAG/CTT (21,323, 4.22%) among the trinucleotide repeats. The most abundant tetranucleotide and pentanucleotide repeats were AAAG/CTTT (4,714, 0.93%) and AAAAG/CTTTT (1,568, 0.31%), respectively. Among the other arthropods, 92 SSRs were detected for the butterfly* Euphydryas editha* [43], 149 for the citrus whitefly,* Dialeurodes citri* [44], 1,249 for* Tenebrio molitor* [45], and 1,100 for the stored-product pest* Liposcelis entomophila* [46]. Variation in the number of SSRs identified could be attributable to the selection criteria used during the analysis. Typically, SSRs sizes of two to six nucleotides in length, with minimum repeat units of six for dinucleotides, five for trinucleotides and tetranucleotides, and four for pentanucleotides and hexanucleotides, have been considered. In another study, only unigene sequences longer than 1 Kb with the above selection criteria were considered for SSR selection [47].

## 4. Conclusions

In this study, the complete transcriptome of the wasp* V. mandarinia* was sequenced using the Illumina HiSeq 4000 NGS platform. The sequences were assembled to meaningful unigene sequences using the* de novo* assembler Trinity and the clustering tool TGICL. The unigene sequences were annotated to publicly available protein sequence databases for putative functional attributes, especially their assignments to GO categories and KEGG biochemical pathways. In addition, we obtained a set of reliable SSR markers from the unigene sequences. This valuable genetic information should be useful for research on functional genes using genomic and proteomic tools. Additionally, markers can be developed to help conserve* V. mandarinia* in its preferred habitat.

## Supplementary Material

Supplementary Figure 1: An overview of transcriptome characterization process in non-model *Vespa mandarinia*.
Supplementary Figure 2(A): Size distribution of *V. mandarinia* contigs after assembly using Trinity *de novo* program.
Supplementary Figure 2(B): Size distribution of *V. mandarinia* unigenes after assembly using Trinity *de novo* program.
Supplementary Table 1: Summary of raw read processing and assembly parameters after Illumina HiSeq 4000 sequencing of *V. mandarinia*. Cutadapt program was used for the thinning and trimming analysis.
Supplementary Table 2: Summary of SSR search in the unigenes of *Vespa mandarinia*.

## Figures and Tables

**Table 1:** Summary statistics of the transcription assembly for *Vespa mandarinia*.

Total number of raw reads	
Number of sequences	60,723,154
Number of bases	9,169,196,254
Total number of clean reads	
Number of sequences	59,184,811
Number of bases	8,297,028,222
Mean length (bp)	140.2
N50 length (bp)	151
GC %	37.93
High-quality reads (%)	97.47 (sequences), 90.49 (bases)
Number of reads discarded (%)	2.53 (sequences), 9.51 (bases)
Contig information	
Total number of contig	147,400
Number of bases	181,439,800
Mean length of contig (bp)	1,230.9
N50 length of contig (bp)	2,578
GC % of contig	35.50
Largest contig (bp)	30,772
Number of large contigs (≥500 bp)	75,428
Unigene information	
Total number of unigenes	66,837
Number of bases	95,657,681
Mean length of unigene (bp)	1,431.2
N50 length of unigene (bp)	3,112
GC % of unigene	35.26
Length ranges (bp)	224–52,946

**Table 2:** The assembled unigenes mapped to the coverage of *Vespa mandarinia* mitochondrial protein coding genes using BLASTN at a cutoff *E*-value of 1*E* − 5.

Q ID	S ID	QLen	HSP	HLen	CovQ	Qx	Qy	Sx	Sy	SLen	CovS
Vm_Uni_01523	*NADH Dehydrogenase subunit 5 (ND5)*	2747	1/2	1680	61.2	181	1860	1680	1	1680	100

Vm_Uni_08557	*NADH Dehydrogenase subunit 3 (ND3)*	9130	1/1	351	3.8	7888	8238	1	351	351	100
	*NADH Dehydrogenase subunit 2 (ND2)*	9130	1/4	1008	11	1543	2550	1008	1	1008	100
	*Cytochrome C oxidase subunit III (COXIII)*	9130	1/1	784	8.6	7032	7815	1	784	784	100
	*Cytochrome C oxidase subunit II (COXII)*	9130	1/1	681	7.5	5297	5977	1	681	681	100
	*Cytochrome C oxidase subunit I (COXI)*	9130	1/2	1536	16.8	3686	5221	1	1536	1536	100
	*ATP Synthase F0 subunit 8 (ATP8)*	9130	1/1	168	1.8	6194	6361	1	168	168	100
	*ATP Synthase F0 subunit 6 (ATP6)*	9130	1/1	666	7.3	6365	7030	1	666	666	100

Vm_Uni_10664	*NADH Dehydrogenase subunit 6 (ND6)*	7998	1/1	537	6.7	4791	5327	537	1	537	100
	*NADH Dehydrogenase subunit 4L (ND4L)*	7998	1/1	297	3.7	5536	5832	1	297	297	100
	*NADH Dehydrogenase subunit 4 (ND4)*	7998	1/1	1313	16.4	5826	7138	1	1313	1317	99.7
	*NADH Dehydrogenase subunit 1 (ND1)*	7998	1/1	951	11.9	2423	3373	1	951	951	100
	*Cytochrome B*	7998	1/1	1143	14.3	3644	4786	1143	1	1143	100

Informative parameters extracted from BLAST hits are: Q ID (query identifier); S ID (subject identifier); QLen (query sequence length); HSP (number of HSPs); CovQ (percent query coverage); Qx and Qy (query coordinators, i.e., from and to); Sx and Sy (subject coordinators, i.e., from and to); SLen (subject length); CovS (percent subject coverage).

**Table 3:** Comparison of the number of unigene annotations obtained from the different protein databases. The number of unigenes hits from locally curable PANM-DB, Unigene, KOG, GO, and KEGG databases, respectively, using BLASTX search (*E*-value ≤ 1*E* − 5).

	All	≤300 bp	300–1000 bp	≥1000 bp
PANM-DB	40,887	3,689	14,531	22,667
Unigene	44,455	4,842	18,298	21,315
KOG	22,390	765	3,790	17,835
GO	15,675	625	2,098	12,952
KEGG	5,111	286	562	4,263
All	51,143	6,221	21,351	23,571

**Table 4:** Summary of top 20 domains predicted in *V. mandarinia* unigene sequences.

IPR accession	Domain short name	Domain description	Unigene
IPR015880	Znf_C2H2	Zinc finger, C2H2-like domain	378
IPR002290	Ser/Thr_dual-sp_kinase	Serine/threonine/dual specificity protein kinase, catalytic domain	250
IPR000504	RRM_dom	RNA recognition motif domain	219
IPR001680	WD40_repeat_dom	WD40 repeat domain	212
IPR003593	AAA+_ATPase	AAA+ ATPase domain	184
IPR027417	P-loop_NTPase	P-loop containing nucleoside triphosphate hydrolase domain	178
IPR002110	Ankyrin_rpt	Ankyrin repeat	163
IPR011701	MFS	Major facilitator superfamily	148
IPR001478	PDZ	PDZ domain	144
IPR000008	C2_dom	C2 domain	119
IPR011989	ARM-like	Armadillo-like helical domain	118
IPR000210	BTB/POZ_dom	BTB/POZ domain	116
IPR001452	SH3_domain	SH3 domain	116
IPR019734	TPR_repeat	Tetratricopeptide repeat	116
IPR000276	GPCR_Rhodpsn	G protein-coupled receptor, rhodopsin-like family	108
IPR001841	Znf_RING	Zinc finger, RING-type domain	92
IPR029058	AB_hydrolase	Alpha/beta hydrolase fold domain	92
IPR001128	Cyt_P450	Cytochrome P450 family	89
IPR001849	PH_domain	Pleckstrin homology domain	89
IPR003591	Leu-rich_rpt_typical-subtyp	Leucine-rich repeat, typical subtype repeat	89

**Table 5:** Distribution of simple sequence repeat (SSR) among the unigenes of *Vespa mandarinia* based on the number of repeat units.

Repeat numbers	Di	Tri	Tetra	Penta	Hexa	Hepta	Octa	Total
3	275239	78234	12632	2814	3053	405	511	372888
4	43886	10895	3176	711	776	72	35	59551
5	12933	4766	1632	325	100	27	10	19793
6	8059	2918	1394	48	87	18	5	12529
7	6168	2096	224	79	75	13	4	8659
8	5218	2082	325	35	32	1	0	7693
9	3823	478	206	37	41	0	1	4586
10	2862	541	132	21	27	1	0	3584
11	2678	440	124	24	11	0	0	3277
12	2175	347	72	15	4	0	0	2613
13	613	306	53	6	5	0	0	983
14	828	263	43	5	2	0	0	1141
15	831	191	35	5	5	0	0	1067
16	664	196	43	3	1	0	0	907
17	659	171	22	1	2	0	0	855
18	539	159	14	0	2	0	0	714
19	504	124	6	0	1	0	0	635
20	395	122	15	0	0	0	0	532
>21	2376	681	34	5	1	0	0	3097
Total	**370450**	**105010**	**20182**	**4134**	**4225**	**537**	**566**	**505104**
