# Supplementary material for: Transcriptome Profile of the Asian Giant Hornet (Vespa mandarinia) Using Illumina HiSeq 4000 Sequencing: De Novo Assembly, Functional Annotation, and Discovery of SSR Markers
Source: Int J Genomics. 2016 Jan 10;2016:4169587. doi: 10.1155/2016/4169587 (PMC4736913; doi:10.1155/2016/4169587)
Supplement: Supplementary file 1 — Supplementary Figure 1: An overview of transcriptome characterization process in non-model Vespa mandarinia. Supplementary Figure 2(A): Size distribution of V. mandarinia contigs after assembly using Trinity de novo program. Supplementary Figure 2(B): Size distribution of V. mandarinia unigenes after assembly using Trinity de novo program. Supplementary Table 1: Summary of raw read processing and assembly parameters after Illumina HiSeq 4000 sequencing of V. mandarinia. Cutadapt program was used for the thinning and trimming analysis. Supplementary Table 2: Summary of SSR search in the unigenes of Vespa mandarinia. [file 4169587.f1.pdf]

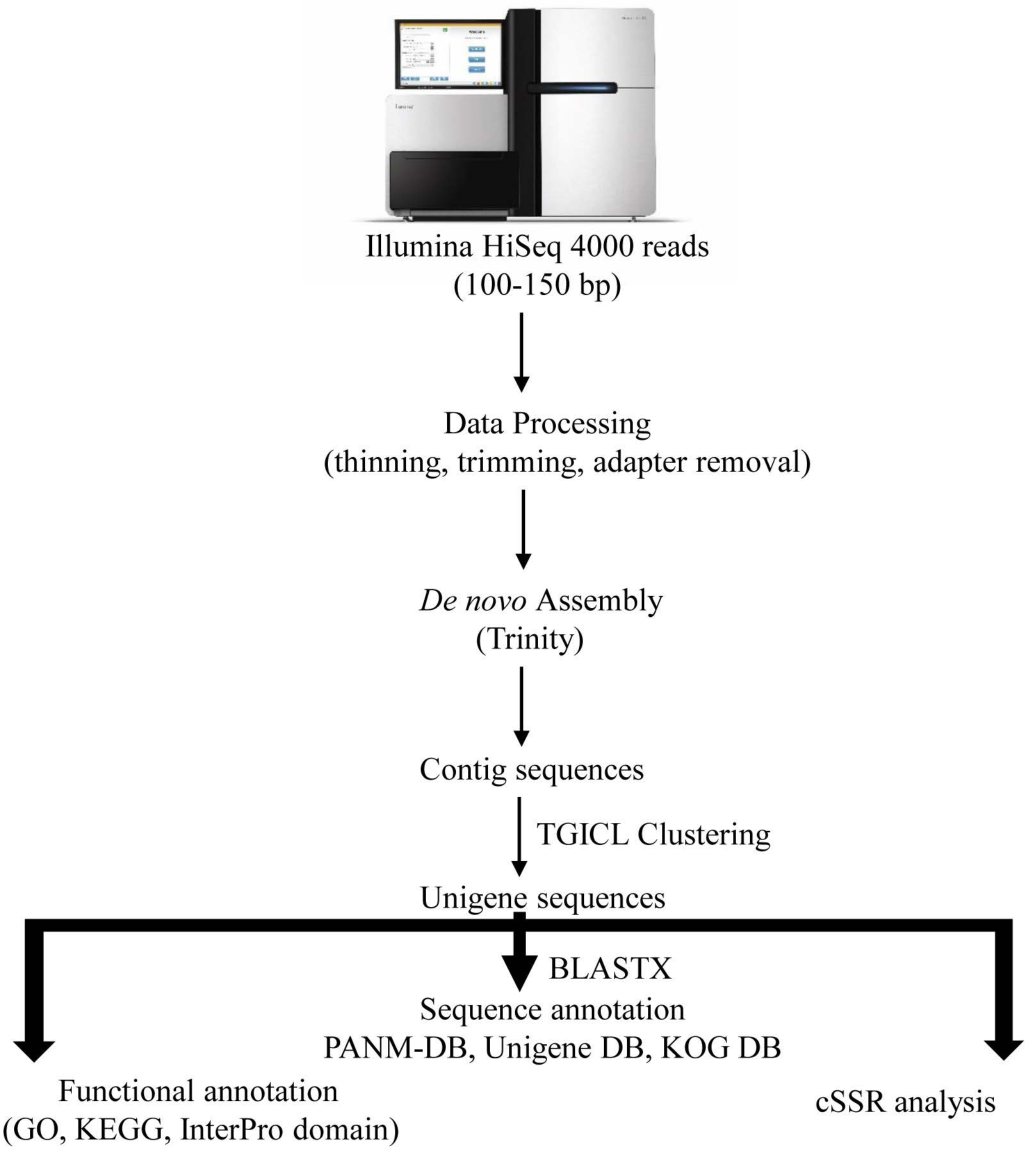
Illumina HiSeq 4000 reads
(100-150 bp)
Data Processing
(thinning, trimming, adapter removal)
De novo Assembly
(Trinity)
Contig sequences
TGICL Clustering
Unigene sequences
BLASTX
Sequence annotation
PANM-DB, Unigene DB, KOG DB
Functional annotation
(GO, KEGG, InterPro domain)
cSSR analysis

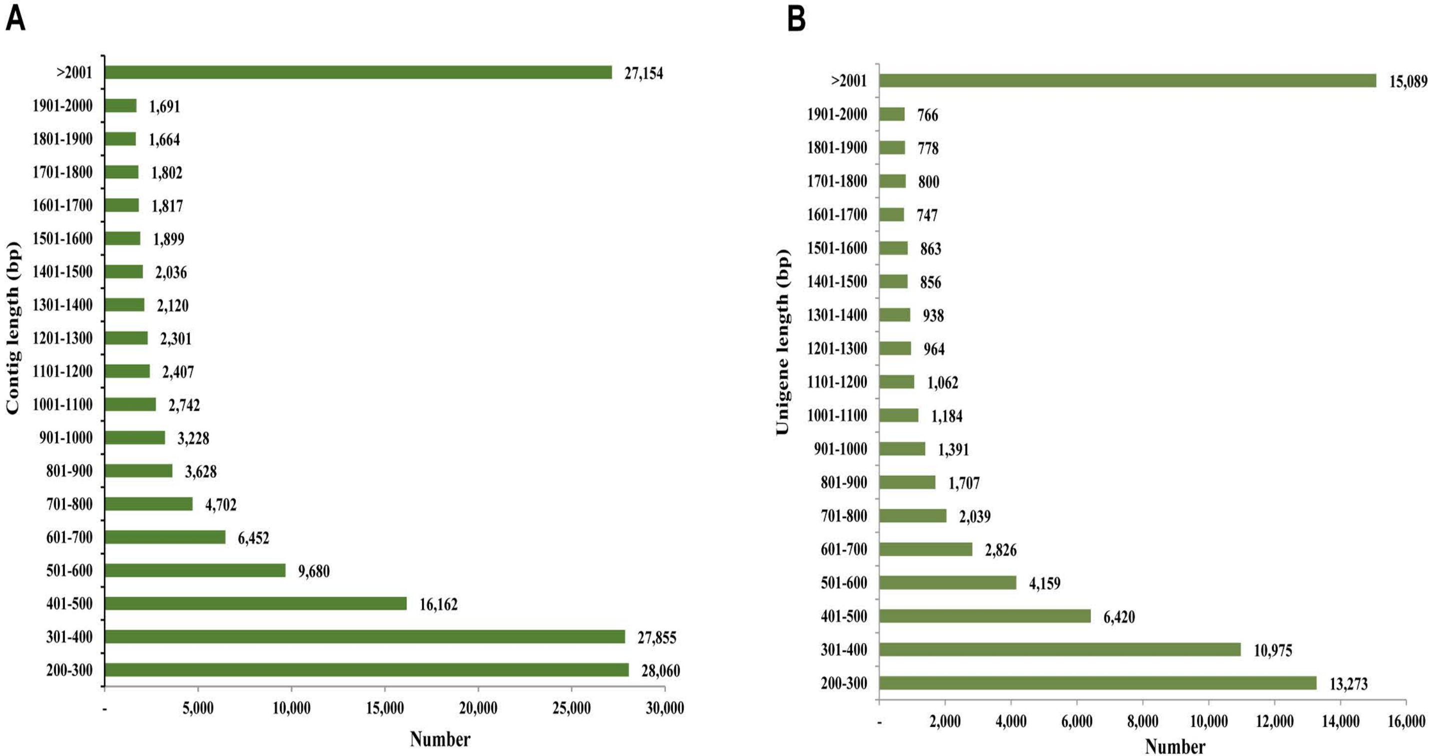

A
Contig length (bp)
Number
>2001
1901-2000
1801-1900
1701-1800
1601-1700
1501-1600
1401-1500
1301-1400
1201-1300
1101-1200
1001-1100
901-1000
801-900
701-800
601-700
501-600
401-500
301-400
200-300
27,154
1,691
1,664
1,802
1,817
1,899
2,036
2,120
2,301
2,407
2,742
3,228
3,628
4,702
6,452
9,680
16,162
27,855
28,060
-
5,000
10,000
15,000
20,000
25,000
30,000
B
Unigene length (bp)
Number
>2001
1901-2000
1801-1900
1701-1800
1601-1700
1501-1600
1401-1500
1301-1400
1201-1300
1101-1200
1001-1100
901-1000
801-900
701-800
601-700
501-600
401-500
301-400
200-300
15,089
766
778
800
747
863
856
938
964
1,062
1,184
1,391
1,707
2,039
2,826
4,159
6,420
10,975
13,273
-
2,000
4,000
6,000
8,000
10,000
12,000
14,000
16,000

Supplementary Table 1.

Summary of raw read processing and assembly parameters after Illumina HiSeq 4000 sequencing of *V. mandarinia*. Cutadapt program was used for the thinning and trimming analyses.

**Data Processing Parameters**	**Read values**
Total number of raw reads	
- Number of sequences	60,723,154
- Number of bases	9,169,196,254
Total read pairs processed	30,361,577
- Read 1 with adapter	2,723,248
- Read 2 with adapter	2,511,316
Pairs written (passing filters)	30,361,577
Total base pairs processed (bp)	9,169,196,254
- Read 1 (bp)	4,584,598,127
- Read 2 (bp)	4,584,598,127
Total written (filtered) (bp)	9,002,691,935
- Read 1 (bp)	4,498,204,748
- Read 2 (bp)	4,504,487,187
Percent of reads after trimming	98.18
Percent of reads discarded	1.82
Average length after trimming (bp)	148.3

Adapter 1 sequence	AGATCGGAAGAGCACACGTCTGAACTCCAGTCAC
Adapter 2 sequence	AGATCGGAAGAGCGTCGTGTAGGGAAAGAGTGTAGATCTCGGTGGTCGCCGTATCATT

Supplementary Table 2.

Summary of SSR search in the unigenes of *V. mandarinia*

**Searching item**	**Numbers**
Total number of sequences examined	66837
Total size of examined sequences (bp)	95657681
Total number of identified SSRs	534922
Number of SSR containing sequences	62522
Number of sequences containing more than 1 SSR	56378
Number of SSRs present in compound formation	275493
**Unit size**	**Number of SSRs**
1	29670
2	370450
3	105010
4	20182
5	4134
6	4225
7	537
8	566
10	148
**Total**	**534922**
